# Associations between Sleep Duration and Anthropometric Indices of Adiposity in Female University Students

**DOI:** 10.3390/ijerph191811681

**Published:** 2022-09-16

**Authors:** Beata Borowska, Agnieszka Suder, Katarzyna Kliś, Iwona Wronka

**Affiliations:** 1 Department of Anthropology, Faculty of Biology and Environmental Protection, University of Łódź, 90-136 Lodz, Poland; 2 Department of Anatomy, University of Physical Education, 31-571 Krakow, Poland; 3 Department of Human Biology, University of Wroclaw, 50-137 Wroclaw, Poland; 4 Laboratory of Anthropology, Institute of Zoology and Biomedical Research, Faculty of Biology, Jagiellonian University, 31-007 Krakow, Poland

**Keywords:** anthropologic indices, sleep duration, BMI

## Abstract

Objectives: To examine associations between sleep duration as well as time of going to sleep and anthropometric indices related to the amount and distribution of adiposity. Material: A total of 969 female university students, aged 19–24 years. Methods: Participants self-reported their sleep duration. Body weight, height, and waist circumference were measured. BMI, WHR and WHtR were calculated. Statistical analyses of results involved logistic regression models. Socioeconomic status and level of stress were added as covariates. Results: In 15% of the sample, sleep was too short (<6 h), and 10% slept too long (>8 h). Compared to women who followed the recommended sleep duration, among short sleepers, both underweight and overweight were more frequent, while long sleepers were more likely to be overweight. A higher prevalence of abdominal obesity and increased risk of metabolic syndrome were observed in both short and long sleepers than in recommended sleepers. Irregular sleep times were connected with higher OR, both for BMI < 18.5 and BMI > 25, for WC > 80, and WHtR below 0.4 and above 0.5. Irregular sleep times also led to an increased risk of metabolic diseases prevalence. Conclusions: Both too long and too short sleep increases the risk of overweight, obesity and abdominal obesity and, as a consequence, the risk of metabolic syndrome in young women.

## 1. Introduction

The optimum daily sleep duration for adults, conferring the greatest health benefits, is thought to range from 7 to 8 h. In practice, actual sleep duration often deviates from the recommended time. According to numerous studies, almost half of the adult population sleep too little (less than 6 h) or too much (more than 9 h) [1,2].

Sleep is part of the basic biological needs of a human. Sleep deprivation brings about much worse consequences than starving, and this is worse when the person is younger [3]. There exist a few theories concerning the role of sleep; however, none of them sufficiently explain the method of all of its biological functions. Sleep deprivation or a complete lack of sleep affect many behavioural and physiological functions [4,5,6]. They cause impairment of alertness, concentration and memory, and may also lead to some metabolic, hormonal and immunological disorders. Chronic sleeplessness contributes to weakening of glucose tolerance; it may also lower concentration of TSH and increase evening concentration of cortisol and sympathetic hyperactivity, which may constitute risk factors of obesity, hypertension and diabetes [5].

According to the latest data gathered by WHO, the prevalence of overweight and obesity has doubled over the last four decades. At the same time, almost half of adults sleep too little—below 6 h a day—or too long—above 9 h daily [1]. Shift work, enforced or long-term shortening of sleep, excessive alertness as well as stress negatively affect quality of life [7]. More and more research is proving that insufficient length and low quality of sleep add to body weight disorders and lead to obesity [8,9,10].

In recent years, much attention has been given to sleep duration and quality’s link with BMI and abnormal body weight. However, existing research results are inconsistent. Some authors have reported a negative correlation between sleep duration and BMI [11,12], while others have indicated a U-shaped relationship [13,14] or no association at all [15,16]. Additionally, only few works have considered students. Research has mainly been conducted among children and in groups of adults of an older age range. Students are a specific research group. They are young people, exposed to very similar factors connected with their work (study) environment and accommodation (dormitories or shared flats). Moreover, the beginning of studies is also the beginning of independent life outside their family home, which is often linked to a change in life conditions and style.

Students are people starting their adulthood, after finishing progressive ontogenesis and before entering the reproductive period. At this stage of biological development, they should reach their peak biological condition. This group should be particularly monitored to detect potential disorders and correct them before inducing more serious health results. This is particularly important in the case of young females as their health condition will affect the health of their offspring.

Taking into consideration both divergences in research results concerning dependences between length of sleep and obesity, and the small number of research studies in the area comprising young adults, it seems justified to conduct such research work.

Apart from sleep length, this study took into account time if sleep. Proper sleep is responsible for maintaining an organism’s homeostasis [17], and also has a regenerative function, but it must fall at an appropriate time during a day [3]. Length, quality, and time of sleep affect circadian rhythm [6,10,18]. The standard time to go to sleep is assumed to be 11:00 p.m., whereas awakening time is 7:00 a.m. [3].

The objective of this work was to determine the relationship between sleep duration as well as going to sleep time and the occurrence of abnormal anthropometric indicators associated with general and abdominal adiposity in female university students. The presented research is to find out whether the negative effects of improper sleep hygiene are noticeable already at a young age.

## 2. Materials and Methods

The study material includes a cross-sectional sample of 969 female students of the Jagiellonian University in Krakow, the University of Physical Education in Krakow, the University of Łódź, and the University of Wrocław. The mean age of the subjects was 22.6 years with a standard deviation of 1.91 years and a median of 22.00 years.

The students were recruited to take part in the study through a direct invitation. On the day of the study, after consulting with the management of the universities, visits were made in all classes. The purpose and course of surveys were explained, and the women were invited to participate in the study. After accepting the invitation, students were directed to a specific room in which they completed research questionnaires, and anthropometric parameters were measured. The study protocol was approved by the Ethics Committee of Jagiellonian University in Krakow. The data were collected following the ethical principles stated in the Declaration of Helsinki. All participants gave their written consent to take part in the study.

Measurements involved stature, body weight, and waist circumference, and were conducted in accordance with the current standards of anthropometric methodology. The obtained data were used to calculate the body mass index (BMI) and the waist-to-height ratio (WHtR). BMI results were divided into three categories:

- Less than 18.5—underweight;
- From 18.5 to 25—normal weight;
- More than 25—overweight and obesity [19].

Waist circumference was divided into two categories, and WHtR into three categories:

- Less than 0.4—deficient abdominal adiposity;
- From 0.4 to 0.5—normal abdominal adiposity;
- More than 0.5—excessive abdominal adiposity [20].

Anthropometric indices were also applied to assess the risk of occurrence of metabolic diseases. This was determined based on combined recommendations of body mass index, waist circumference and/or WHtR, in accordance with the following criteria [19,21]:

- Low risk—BMI < 25, waist circumference < 80, and WHtR > 0.5;
- Moderate risk—BMI < 25, waist circumference > 80, and/or WHtR > 0.5;
- High risk—BMI > 25, waist circumference > 80, and WHtR > 0.5 [19,21].

The applied study tool was a questionnaire, which was filled in anonymously by the students. A small briefing was given to the students to explain the terminology used in the questionnaire. Reliability and validity of the questionnaire were checked by the test–retest technique. The questionnaire contained items concerning sleep duration, self-assessment of one’s general health, and the occurrence of chronic diseases. Students reporting chronic diseases were excluded from further study. Additionally, the data of women who were pregnant or having children were not considered.

Sleep duration was obtained by students’ self-report in response to the questionnaire item, “How much sleep do you usually get at night on weekdays or workdays?” When the examined person gave a range of two hours, e.g., 6–7, the average value from this range was entered. If the given range exceeded two hours, it was coded as irregular sleep duration. The questionnaire also included the question “During the past month, what time did you usually go to bed/wake up on weeknights/weekends?” This allowed for additional specification of sleep duration for weeknights and weekends as the difference between bed- and waking time. There was no discrepancy in sleep duration determined on the basis of the first and second questions in any case.

Socioeconomic status (SES), included in the analysis as covariates, was determined based on variables considered reliable indicators of living conditions and lifestyle in Poland and Europe: degree of urbanization of place of residence, parental educational attainment, number of children in the family and family financial status. The following categories were established: for place of residence, a village, a town (up to 100,000 inhabitants), and a city (more than 100,000 inhabitants); for parents’ education level, primary and vocational, secondary, and university; for number of siblings, 0, 1, 2, 3, and more; for material conditions, very good, good, average, below average, variable, and difficult to determine. Only one person assessed the economic situation in her home as “changeable and/or difficult to assess”, so that category was omitted. Principal components analysis (PCA) was applied to establish an SES evaluation index and, based on the value of the first component, the subjects were qualified as belonging to families of low, average, or high SES. Additionally, the students were asked to assess the level of stress in their current life situation.

### Statistical Methods

Statistical analyses of the results were performed with the use of the statistical software Statgraphics Cent. 18 and involved logistic regression models. SES and the level of stress were added as covariates. Odds ratios (OR) and 95% confidence intervals (CI) were presented.

Significance in all statistical tests was set at the level of at least *p* < 0.05.

## 3. Results

### 3.1. Characteristics of the Subjects’ Sleep Duration

Mean sleep duration in the study group was 6.9 h with a standard deviation of 1.25 h and a median of 7.0 h. Reported minimum and maximum sleep durations were 4.0 h and 13.0 h, respectively.

Sleep duration was divided into three categories:

- Short—less than 6 h;
- Normal—from 6 to 8 h;
- Long—more than 8 h.

The majority of subjects reported normal sleep duration (74.8%), with 14.8% reporting a short and 10.4% a long sleep duration.

The highest percentage of the examined females declared average times of going to sleep (67.3%); fewer females went to sleep before 10:00 p.m. (6.9%) and after midnight (20.0%). Irregular times of going to sleep were reported by the lowest number of participants (5.8%). Most females declared early wakening times (41.8%); slightly fewer woke up between 7:00 and 8:00 a.m. (31.5%) and after 8:00 a.m. (21.8%). The lowest number of them woke up at irregular times (4.9%).

### 3.2. Anthropometric Characteristics of the Subjects

The majority of subjects had normal body weight (69.4%), with 12.2% being underweight and 18.4% overweight or obese. Normal abdominal adiposity was found in 68.5% of the subjects, deficient adiposity in 19.2%, and excessive adiposity in 12.3%. Most of the studied females did not feature an elevated risk of metabolic diseases, while 10.1% were classified in the moderate risk group and 13.0% in the high-risk group.

### 3.3. Relationships between Sleep Duration and Anthropometric Indicators

The risk of underweight and overweight was much higher among short sleepers as compared to normal sleepers, while long sleepers were more likely to be overweight as compared to subjects sleeping 6–8 h/day (Table 1).

Both short and long sleepers exhibited a greater likelihood of excessive abdominal adiposity (WC > 80 cm) than normal sleepers. A similar relationship was found for WHtR. In that case, the category of “deficient central adiposity” was used in addition to abdominal obesity. The group of short sleepers revealed higher OR of both deficient and excessive abdominal adiposity as compared to females sleeping 6–8 h/day. In turn, the OR of excessive abdominal adiposity in long sleepers was twice as high as in normal sleepers (Table 1).

The last of the analysed relationships was the association between sleep duration and the risk of metabolic diseases. The risk of metabolic diseases was the lowest among normal sleepers, slightly higher among short sleepers, and by far the highest among long sleepers (triple the value for females sleeping 6–8 h/day) (Table 1).

### 3.4. Relationships between Hours of Sleep and Anthropometric Indicators

Next, correlations between times of going to sleep and risks of occurrence of underweight, overweight, obesity, abdominal obesity and metabolic diseases were investigated. It was observed that women with irregular times of going to sleep are at risk of disorder in the amount of adipose tissue. Irregular sleep times were connected with higher OR, both for BMI < 18.5 and BMI > 25, for WC > 80, and WHtR below 0.4 and above 0.5. Irregular sleep times also led to an increased risk of metabolic diseases (Table 2).

## 4. Discussion

Mean sleep duration in the study group was 6.9 h, with 15% of the students reporting sleep times below 6 h, and 10% above 8 h. Similar results have been obtained in other Polish surveys involving college students [2].

The presented results as well as findings from other authors show that increasing numbers of people tend to sleep too little or their sleep quality is insufficient to ensure appropriate rest [1,7]. Consequently, a growing body of research has been dedicated to the identification of the underlying causes and effects of this phenomenon [8,9].

Numerous studies have explored associations between sleep duration and abnormal adiposity. For instance, Sayón-Orea et al., who examined a Mediterranean population, indicated that short sleep duration (<5 h) entails a higher risk of obesity [22]. Similar findings have been reported from a Japanese study: short sleep duration was found to be related not only to the overall amount of adipose tissue, but also its distribution. Chronic sleep deprivation has been shown to be a risk factor for the most health-damaging form of obesity—abdominal obesity [23]. It has also been found that, in children and adolescents, insufficient sleep duration is associated with higher BMI values and a greater prevalence of overweight and obesity [9]. An extensive literature review by Patel and Hu has revealed that too little sleep leads to a greater risk of obesity in children and young adults as compared to older individuals, which would suggest that the relationship between sleep duration and body weight diminishes with age [24], which has been corroborated by other scholars [12].

In turn, a survey of over 2300 students enrolled at the University of Zagreb in 2017 showed statistically significant relationships between time spent in bed and sleep quality associated with overweight/obesity. Both short and long time spent in bed as well as poor sleep quality contribute to a higher likelihood of overweight/obesity in young adults [25]. A significant relationship between sleep quality and general obesity and high adiposity has been noted in a German investigation by Rahe and collaborators [26]. Finally, evidence from a population-based cohort study has shown that poor sleep quality is associated with weight gain [27]. Similar results have been obtained by Salarinia et al. [28].

The current findings reveal statistically significant relationships between sleep duration and adiposity. As compared to normal sleepers, short sleepers are more often underweight or overweight, while long sleepers tend to suffer from overweight. This is also true of central adiposity. Short sleepers have a greater propensity for deficient or excessive abdominal adiposity, and long sleepers are twice as likely as normal sleepers to have abdominal adiposity. This is consistent with a Swedish study of 5000 females, which has revealed that both short and long habitual sleep durations are correlated with a greater occurrence of general obesity as compared to normal sleep duration, while habitual long sleep duration is a risk factor for central adiposity [14]. Similar findings from other authors suggest that both too little and too much sleep increase the risk of general obesity [1,29,30] as well as central obesity [31,32].

In the scientific literature, studies can be found not only concerning the relation between sleep duration and the amount of adipose tissue, but also clarifying the relationship of the mechanism. Too short sleep results in a lower level of leptin and simultaneous growth in ghrelin level [8,33,34,35,36]. Consequently, this may lead to obesity and the development of metabolic disorders [9,37].

Apart from leptin and ghrelin, other hormones may also influence the growth of body weight in response to sleep deficiency. Glucagon-like peptide (GLP-1) inhibits emptying of the stomach and promotes a feeling of satiety. In females, the level of GLP-1 is lowered by short sleep, which favours overeating and leads to a growth in body weight [1,38]. The endocannabinoid system rapidly increases appetite and food intake by a hedonic motivation to eat. Sleep deficiency increases activity of neurons engaged in the reward system. Studies indicate that it may be one of the paths through which an insufficient length of sleep favours excessive eating and leads to obesity [9,39,40,41].

Sleep shortening decreases peripheral sensitivity to insulin and promotes hunger. Maintaining a lower activity of insulin and increased intake of calories may result in body weight growth and, consequently, the development of obesity [8,36,42].

A significant role in inducing obesity in people with sleep disorders is played by stress. Insufficient sleep duration decreases resistance to stressful situations, and one of the most common reactions in such circumstances is increasing the amount of consumed food. Chronic and uncontrolled stress may lead to metabolic disorders and cause increased appetite [43,44].

In obese patients, sleep disorders occur twice more frequently than in people with normal body weight [10]. Therefore, insufficient body weight may be both a consequence and a cause of obesity [43,45]. Obesity affects the length and quality of sleep, suppressing circadian hormone variability [42]. At the same time, improper sleep is a potential risk factor of body weight growth and obesity development through a negative influence on metabolic changes [42,43].

Despite a wide-ranging analysis of mechanisms connecting short sleep with obesity, there are significantly fewer reviews that link it to long sleep. Meanwhile, long sleep is connected with a risk of obesity development that is 21% higher than in people sleeping 7–8 h daily [46]. People who sleep for a long time most frequently use sleeping pills, which may demonstrate problems with falling or staying asleep. A longer sleep may also be a consequence of a bad quality of sleep [1]. According to Lau et al., people with higher BMI sleep longer and the higher the body weight, the worse the quality of sleep [43]. Habitual short or long sleep may be partially caused by obesity-related illnesses such as depression or hypertension, which negatively affect sleep quality [1]. Additionally, a sedentary lifestyle combined with a lack of physical activity may contribute to obesity in people with a long sleep duration [1]. Moreover, eating patterns similar to those of people with short sleep in the form of irregular meals and a higher number of snacks daily increase the risk of obesity prevalence in people sleeping ≥9 h [47].

The majority of works published in the scientific literature indicate the influence of improper sleep length on the prevalence of obesity. However, a reverse correlation cannot be excluded. Excessive amount of adipose tissue causes metabolic changes in the body, which subsequently lead to the occurrence of many diseases. Health disorders related to obesity may have an influence on improper sleep length [1]. People who do not care about following a proper lifestyle do not pay attention to dietary habits, engage in a sufficient level of physical activity and take care of sleep hygiene, which results in obesity and sleep disorders. Due to the fact that most of the research in this area were of a cross-sectional nature, explaining the relation mechanism between length and quality of sleep and the occurrence of obesity is hindered.

The present study has certain limitations. First, the data on sleep duration were obtained using a self-report questionnaire, so they may be susceptible to subjective errors of judgment. Second, the study did not take into account covariate predictors such as physical activity, diet, substance use, as well as sociopsychological factors, which may have directly affected the results. Finally, the study was limited to females aged 20 to 27 years, so the identified relationships may differ from those prevalent among the general population.

## 5. Conclusions

The presented results and literature data indicate that a substantial proportion of young people sleep too little or too much. A strong correlation has been found between sleep duration, the occurrence of sleep disturbances, and abnormal body weight and, consequently, obesity-related diseases. The typical lifestyle of young people, characterized by high intensity and haste in performing tasks linked to social functioning, may lead to a further reduction in average sleep duration and a deterioration in sleep disturbances. Thus, it is necessary not only to continue research on these problems, but also implement preventive initiatives. Good sleep is indispensable for normal functioning, and abnormal sleep duration may have serious consequences for the function of numerous organs and tissue systems.

It should be emphasised that the present work shows relations between length and time of sleep and anthropometric indices. Although several works indicate that they are connected with the occurrence of metabolic diseases and are recommended to apply in cross-sectional studies by many scientific and medical associations, including WHO, they only determine the risk of metabolic syndrome and not its actual occurrence [48]. Not every person with increased values of the indices is affected by metabolic disorders. However, there is a high probability based on the results of scientific works that people characterised by higher values of these indices will suffer from these disorders in the future if these indices are not lowered. Therefore, following the rule that it is better to prevent than cure, the prevention of metabolic diseases should take into account not only dietary habits and physical activity, but also other aspects of a healthy lifestyle, mainly including hygiene of sleep.

## Figures and Tables

**Table 1:** The risk (OR and 95% CI) of underweight and overweight and abdominal obesity in relation to sleep duration.

	Sleep Duration			
Anthropometric Indices Category	Short	Normal	Long	*p*-Values
BMI < 18.5	1.98 (1.32–2.04)	1	0.49 (0.13–1.01)	0.01 **
BMI > 25	1.97 (1.38–2.16)	1	2.34 (1.60–2.69)	0.006 **
WC > 80	1.82 (1.06–2.08)	1	2.23 (1.52–2.03)	0.01 **
WHtR < 0.4	1.73 (1.21–2.17)	1	0.71 (0.51–0.94)	0.012 *
WHtR > 0.5	1.84 (1.22–2.20)	1	2.29 (1.86–2.59)	0.008 **
Overweight and abdominal obesity	1.21 (0.81–1.34)	1	3.01 (2.41–3.40)	0.000 ***

*p*-values based on logistic regression model, adjusted for confounding factors: stress level and socio-economic factors. * significance at *p* < 0.05, ** significance at *p* < 0.01, *** significance at *p* < 0.001.

**Table 2:** The risk (OR and 95% CI) of underweight and overweight and abdominal obesity in relation to times of going to sleep.

	Times of Going to Sleep				
Anthropometric Indices Category	Early	Normal	Late	Irregular	*p*-Values
BMI < 18.5	1.16 (1.02–1.94)	1	0.62 (0.43–1.29)	1.80 (1.05–2.18)	0.012 *
BMI > 25	1.19 (1.04–1.99)	1	1.14 (1.01–1.62)	1.91 (1.12–2.21)	0.011 *
WC > 80	1.12 (0.64–1.11)	1	1.12 (0.90–1.91)	1.72 (1.04–2.11)	0.015 *
WHtR < 0.4	1.04 (0.52–1.86)	1	0.81 (0.41–0.89)	1.74 (0.52–2.06)	0.012 *
WHtR > 0.5	1.03 (0.39–1.95)	1	1.12 (0.88–1.98)	1.73 (0.39–2.05)	0.015 *
Overweight and abdominal obesity	1.11 (0.62–1.61)	1	1.14 (1.02–1.65)	1.81 (0.62–2.06)	0.013 *

*p*-values based on logistic regression model, adjusted for confounding factors: stress level and socio-economic factors. * significance at *p* < 0.05.

## Data Availability

The data presented in this study are available upon request from the corresponding author. The data are not publicly available due to privacy.
